# A review of policies and programmes for human organ and tissue donations and transplantations, WHO African Region

**DOI:** 10.2471/BLT.19.236992

**Published:** 2020-04-28

**Authors:** André Loua, Margot Feroleto, Aissatou Sougou, Ossy Muganga Julius Kasilo, Jean Baptiste Nikiema, Walter Fuller, Stanislav Kniazkov, Prosper Tumusiime

**Affiliations:** a World Health Organization Regional Office for Africa, Cite du Djoue, P.O. Box 06, Brazzaville, Congo.; b Department of International Health, Georgetown University, Washington, DC, United States of America.

## Abstract

Several resolutions, endorsed by the World Health Assembly and the United Nations General Assembly, articulate the need to improve the availability, quality and safety of organ and tissue donation and transplantation, as well as to prevent and combat trafficking in human organs. Here we assessed the implementation of these resolutions pertaining to organ and tissue donations and transplantations by sending out a questionnaire to all 47 countries in the World Health Organization African Region. From 33 countries that provided data, we identified several obstacles and challenges. Compared to other regions, there are very limited data on organ donation and transplantation. Most countries are lacking legal and regulatory frameworks, since they did not yet establish a specific or comprehensive legislation covering donation and transplantation of human organs and tissues. Countries also have a poor national capacity to perform organ and tissue transplantations and the organization and management of national programmes are weak. Funding, both from domestic and external sources, is insufficient to implement effective transplantations programmes and patients have inadequate financial protection. To address these challenges, we propose that countries and partners should develop and implement policies, strategies, plans and regulatory frameworks for all aspects of organ and tissue donations and transplantations, including fighting against organ trafficking and transplant tourism. Where donation and transplantation programmes exist, stakeholders should develop the skills of human resources, adopt technical standards and quality management procedures to improve donation and transplantation of human organs and tissues.

## Résumé

De nombreuses résolutions approuvées par l'Assemblée mondiale de la Santé et l'Assemblée générale des Nations Unies ont souligné le besoin d'améliorer la disponibilité, la qualité et la sécurité des dons et de transplantations d'organes et de tissus humains, de même que celui d'assurer la prévention et la lutte contre le trafic d'organes. Dans ce document, nous avons étudié la mise en œuvre de ces résolutions relatives aux dons et transplantations d'organes et de tissus, par le biais d'un questionnaire envoyé aux 47 pays appartenant à la région Afrique de l'Organisation mondiale de la Santé. Nous avons identifié de nombreux obstacles et défis dans les 33 pays qui nous ont transmis des données. Par rapport à d'autres régions, il existe très peu d'informations à ce propos. La plupart des pays ne possèdent pas de cadres juridiques et réglementaires car ils n'ont pas encore établi de législation spécifique ou exhaustive couvrant les dons et transplantations d'organes et de tissus. Certains manquent également de moyens au niveau national pour réaliser des greffes d'organes et de tissus, tandis que leur organisation et leur gestion des programmes nationaux sont inadaptées. Tant les fonds provenant de l'intérieur que ceux fournis par l'extérieur ne permettent pas d'instaurer des programmes de transplantation efficaces. Enfin, les patients ne bénéficient pas d'une protection financière suffisante. Afin de pouvoir relever ces défis, nous proposons que ces pays et leurs partenaires développent et appliquent des politiques, stratégies, projets et règles pour tous les aspects liés aux dons et transplantations d'organes et de tissus, y compris la lutte contre le trafic d'organes et le tourisme de la transplantation. Et là où des programmes de dons et de transplantations existent, les intervenants devraient acquérir des compétences en ressources humaines, mais aussi adopter des normes techniques et des procédures de gestion de la qualité afin d'optimiser les dons et transplantations d'organes et de tissus.

## Resumen

Diversas resoluciones que la Asamblea Mundial de la Salud y la Asamblea General de las Naciones Unidas aprobaron articulan la necesidad de mejorar la disponibilidad, la calidad y la seguridad de la donación y el trasplante de tejidos y órganos, así como de prevenir y combatir el tráfico de órganos humanos. En el presente documento se evalúa la implementación de estas resoluciones relacionadas con la donación y el trasplante de tejidos y órganos por medio del envío de un cuestionario a los 47 países de la Región de África de la Organización Mundial de la Salud. De los 33 países que suministraron los datos, se identificaron varios obstáculos y desafíos. En comparación con otras regiones, existen muy pocos datos sobre la donación y el trasplante de órganos. La mayoría de los países carecen de marcos normativos y legales, ya que todavía no han establecido una legislación específica o integral que aborde la donación y el trasplante de tejidos y órganos humanos. Los países también tienen una capacidad nacional deficiente para realizar los trasplantes de tejidos y órganos, además de que la organización y la gestión de los programas nacionales son débiles. El financiamiento, tanto de fuentes nacionales como internacionales, es insuficiente para implementar programas de trasplantes efectivos y los pacientes tienen una protección financiera inadecuada. Para hacer frente a estos desafíos, se propone que los países y los socios elaboren e implementen políticas, estrategias, programas y marcos normativos de todos los aspectos de la donación y el trasplante de tejidos y órganos, incluida la lucha contra el tráfico de órganos y el turismo de trasplantes. Cuando existan programas de donación y trasplante, las partes interesadas deberían desarrollar las habilidades de los recursos humanos y adoptar estándares técnicos y procedimientos de gestión de calidad para mejorar la donación y el trasplante de tejidos y órganos humanos.

## ملخص

إن العديد من القرارات التي أقرتها جمعية الصحة العالمية والجمعية العامة للأمم المتحدة، توضح الحاجة إلى تحسين توافر ونوعية وسلامة التبرع بالأعضاء والأنسجة وزرعها، فضلاً عن منع ومكافحة الاتجار بالأعضاء البشرية. قمنا هنا بتقييم تنفيذ هذه القرارات المتعلقة بالتبرعات بالأعضاء والأنسجة وزرعها، وذلك عن طريق إرسال استبيان إلى جميع الدول الـ 47 في المنطقة الأفريقية التابعة لمنظمة الصحة العالمية. ومن بين 33 دولة قامت بتقديم البيانات، قمنا بتحديد عقبات وتحديات عديدة. ومقارنة بالمناطق الأخرى، هناك بيانات محدودة للغاية عن التبرع بالأعضاء وزرعها. تفتقر معظم الدول إلى أطر العمل القانونية والتنظيمية، لأنها لم تضع بعد تشريعات محددة أو شاملة تغطي التبرع بالأعضاء والأنسجة البشرية وزرعها. كما أن الدول لديها قدرة وطنية ضعيفة على إجراء عمليات زرع الأعضاء والأنسجة، كما أن تنظيم البرامج الوطنية وإدارتها يتسم بالضعف. كذلك فإن التمويل، سواء من المصادر المحلية والخارجية، غير كاف لتنفيذ برامج زرع فعالة، كذلك فإن المرضى يعانون من حماية مالية غير مناسبة. ولمواجهة هذه التحديات، فإننا نقترح أنه يجب أن تقوم الدول والشركاء بوضع وتنفيذ سياسات واستراتيجيات وخطط وإطار تنظيمي لجميع جوانب التبرع بالأعضاء والأنسجة وزرعها، بما في ذلك مكافحة الاتجار بالأعضاء وسياحة زرع الأعضاء. وحيثما توجد برامج للتبرع بالأعضاء وزرعها، يجب على أصحاب المصلحة تطوير مهارات الموارد البشرية، وانتهاج المعايير الفنية، وإجراءات إدارة الجودة، بهدف تحسين التبرع بالأعضاء والأنسجة البشرية وزرعها.

## 摘要

世界卫生大会和联合国大会通过的几项决议明确指出，需要改进器官和组织捐献与移植的供给情况、质量和安全，以及预防和打击人体器官贩运。在此，我们向世界卫生组织非洲地区的 47 个国家都发送了调查问卷，评估了这些有关器官和组织捐献与移植的决议的执行情况。在提供数据的 33 个国家中，我们发现了一些障碍和挑战。与其他地区相比，关于器官捐献与移植的数据非常有限。大多数国家缺乏法律和监管框架，因为他们尚未设立涵盖捐献与移植人体器官和组织的具体法律或全面法律。各国进行器官和组织移植的能力也很差，国家计划的组织和管理薄弱。来自国内外的资金不足以实施有效的移植计划，并且患者的经济保障不足。为了应对这些挑战，我们建议各国与合作伙伴应针对器官和组织捐献与移植的各个方面制定并实施政策、战略、计划和监管框架，包括打击器官贩运和移植旅游。在存在捐献与移植计划的地方，利益相关者应开发人力资源技能，采用技术标准和质量管理程序，以改善人体器官和组织的捐献与移植。

## Резюме

В ряде резолюций, одобренных Всемирной ассамблеей здравоохранения и Генеральной ассамблеей Организации Объединенных Наций, подчеркивается необходимость повышения доступности, качества и безопасности донорства и трансплантации органов и тканей, а также предотвращения нелегальной торговли человеческими органами и борьбы с ней. В данном документе авторы оценивают осуществление упомянутых резолюций, касающихся донорства и трансплантации органов и тканей, на основе анкетного опроса, проводившегося во всех 47 странах Африканского региона Всемирной организации здравоохранения. На основании данных, полученных из 33 стран, авторы определили ряд препятствий и проблем. В сравнении с другими регионами данные по донорству и трансплантации органов здесь весьма ограниченные. В большинстве стран отсутствует юридическая и правовая база, поскольку они до сих пор не приняли специального или всеобъемлющего законодательства, охватывающего вопрос донорства и трансплантации человеческих органов и тканей. Кроме того, страны обладают слабым потенциалом для проведения операций по трансплантации органов и тканей, а соответствующие национальные программы нуждаются в совершенствовании организации и управления. Финансирование, как внутреннее, так и внешнее, является недостаточным для осуществления эффективных программ по трансплантации, а пациенты не имеют достаточной финансовой защиты. Для решения этих проблем мы предлагаем странам и партнерам разработать и внедрить политики, стратегии, планы и нормативно-правовую базу для всех аспектов донорства и трансплантации органов и тканей, включая борьбу с незаконной торговлей человеческими органами и трансплантационным туризмом. Там, где программы донорства и трансплантации существуют, заинтересованные стороны должны развивать навыки людских ресурсов, внедрять соответствующие технические стандарты и процедуры управления качеством с целью совершенствования процесса донорства и трансплантации человеческих органов и тканей.

## Introduction

Most countries in the World Health Organization (WHO) African Region are experiencing a rapid increase in noncommunicable diseases.^1^^–^^4^ This increase could have a devastating effect on a region with significant resource constraints, because of the costly and resource demanding health-care provision needed to treat many of these diseases. For example, some of these diseases can cause end-stage organ failures and the standard treatment for such indication is donation of organ(s) or tissue(s) for transplantation. A treatment is not only costly, but also requires skilled health professionals, infrastructure, equipment and legal frameworks. The need for appropriate treatment interventions for organ failures in the region is underscored by the high prevalence of chronic kidney disease, which often leads to lethal kidney failure if untreated, in the general population. Estimates from a systematic review showed that between 2012 and 2016, the prevalence in adults varied from 10.4% in southern Africa, to 14.4% in eastern Africa, 16.0% in central Africa and 19.8% in western Africa.^5^ Deaths caused by renal failure in the region are also high. For example, a 10-year audit of a hospital in Cameroon showed a high proportion (44.9%; 297/661) of people on maintenance haemodialysis had died.^6^

In recent years, human organ transplant outcomes have improved due to better surgical techniques, organ preservation, immunosuppression and antimicrobial therapies.^7^ However, for the subset of the African population, who might be considered medically suitable for transplantation, this treatment is still inaccessible. The lack of transplant specialists, health facilities, capacity to achieve acceptable graft outcomes, as well as the affordability of surgery and subsequent lifelong immunosuppression treatment for the patients are major barriers.^8^ Furthermore, patients’ cultural and religious attitudes towards organ donation and trust in the health system may affect the demand for transplantation.

The World Health Assembly (WHA) has adopted two resolutions and a guidance document regarding the availability, safety and appropriate use of organs and tissues: the resolutions on *Human organ and tissue transplantation* (WHA57.18 and WHA63.22);^9^^,^^10^ and the accompanying document *WHO guiding principles on human cell, tissue and organ transplantation*.^11^ In addition, the United Nations (UN) General assembly has adopted the resolution *Strengthening and promoting effective measures and international cooperation on organ donation and transplantation to prevent and combat trafficking in persons for the purpose of organ removal and trafficking in human organs*.^12^ These resolutions and guiding principles urge Member States to take provisions for the availability and safety of organ and tissue donations and transplantations.^9^^–^^11^ Furthermore, the Declaration of Istanbul on Organ Trafficking and Transplant Tourism recommends that all countries need a legal and professional framework to govern organ donation and transplantation activities, as well as a transparent regulatory oversight system that ensures donor and recipient safety and the enforcement of standards and prohibitions on unethical practices.^13^ At the regional level, the Abuja Declaration gives recommendations regarding preventive activities and transplantation for end-stage renal diseases in Africa.^14^

Organ transplantations have been done in the African Region since 1967, when the first heart transplant ever was performed in South Africa.^15^ Since then, other countries in the region have established functional organ transplant programmes. In these countries, there is at least one kidney transplant centre with the capacity to perform kidney transplantation and post-transplant management of recipients within the country’s borders.^16^ Data from Nigeria show that between 2000 and 2010, a total of 143 kidney transplantations from living donors were performed in five transplants centres,^17^ while data from Ghana show that 17 kidney transplants from living donors were performed between 2008 and 2014.^18^ In Ghana, a national registry for end-stage kidney disease and kidney transplantation was established in 2015.^18^ In 2013, the South African Transplant Society invited 10 countries from sub-Saharan African (Cameroon, Ethiopia, Ghana, Kenya, Malawi, Nigeria, Rwanda, Senegal, Sudan, Tunisia, and Zambia), which had the need and ability to develop a transplant programme, to attend a Global Alliance on Transplantation meeting in Durban, South Africa. Of the invited countries, five countries (Ghana, Kenya, Nigeria, Sudan and Tunisia) performed living-related donor transplantation in 2012. South Africa was the only country where both living and deceased donor kidney transplantations have been performed.^19^

Here we assess the level of implementation of the resolutions adopted by WHA^9^^,^^10^ and the UN General Assembly,^12^ pertaining to organ and tissue donations and transplantations in all 47 countries in the WHO African Region. We also propose actions to be taken by countries and partners in the region to strengthen their capacity of performing donations and transplantation of human organs and tissues.

## General overview

We assessed the situation of organ and tissue donations and transplantations through a survey covering all of the 47 countries in the WHO African Region. From September 2016 to December 2018, we sent out a questionnaire by email to relevant authorities in each country. The questionnaire covered legal, organizational and financial topics regarding transplant centres and activities. Respondents from 33 countries filled out the questionnaire: six (18.2%) countries from central Africa (Angola, Burundi, Chad, Cameroon, Congo and Gabon), 15 countries (45.5%) from Eastern and Southern Africa (Comoros, Eritrea, Eswatini, Ethiopia, Kenya, Madagascar, Mauritius, Mozambique, Namibia, Rwanda, Seychelles, South Sudan, United Republic of Tanzania, Uganda and Zimbabwe) and 12 countries (36.3%) from Western Africa (Algeria, Benin, Burkina Faso, Cabo Verde, Côte d'Ivoire, Ghana, Guinea, Guinea Bissau, Mali, Nigeria, Senegal and Sierra Leone). Three of the responding countries (Ethiopia, Kenya and Nigeria) were used to provide data to the Global Observatory on Donation and Transplantation. For the 14 countries that did not fill out the questionnaire, only South Africa was providing its data to the global observatory on a regular basis, while respondents of the other countries lacked knowledge and/or ownership of transplant programmes in their countries.

We identified several challenges in organ and tissue donations and transplantations that countries in the African Region are facing. Examples of these challenges are: (i) insufficient legal requirements and weak regulatory frameworks; (ii) lack of access to transplantation centres due to the absence of appropriate infrastructure; (iii) insufficient technical expertise, including competent human resources and technology; (iv) unavailability for immunosuppressive agents; and (v) lack of funding for such programmes.^8^^,^^18^^,^^20^^,^^21^ Table 1 summarizes the situation of organ and tissue donations and transplantations in the assessed countries.

**Table 1:** Situation of organ donation and transplantations in the WHO African Region, September 2016 to December 2018

Indicator	No.	Countries
**Countries with functional transplantation programmes**		
Functional transplantation programmes from living donors	7	Algeria, Côte d’Ivoire, Ethiopia, Kenya, Namibia, Nigeria, Uganda
**No. of transplant centres in the region**		
Kidney centres	35	Algeria, Côte d’Ivoire, Ethiopia, Kenya, Namibia, Nigeria, Uganda
Corneal centres	17	Algeria, Kenya
Bone marrow centres	5	Algeria
Liver centres	3	Algeria
Heart centres	2	Namibia, Uganda
**Countries having legal requirements**		
Legal requirements in place covering organ donations and/or transplantations	13	Algeria, Burkina Faso, Comoros, Côte d'Ivoire, Ethiopia, Kenya, Mauritius, Namibia, Nigeria, Rwanda, Senegal, Uganda, Zimbabwe
Governments intended to adopt new legal requirements	8	Cameroon, Chad, Eswatini, Ghana, Guinea, Madagascar, Mali, Mozambique
No legislations in place	12	Angola, Benin, Burundi, Cabo Verde, Congo, Eritrea, Gabon, Guinea Bissau, Seychelles, Sierra Leone, South Sudan, United Republic of Tanzania
Legal requirements in place to inform living donors on the risks of the operation	10	Algeria, Comoros, Ethiopia, Kenya, Mali, Nigeria, Rwanda, Senegal, Seychelles, Uganda
Legal restrictions on the coverage of donation costs for living donors	4	Comoros, Mali, Rwanda, Senegal
Legal requirement to follow-up on the outcomes of living donors	5	Algeria, Ethiopia, Mali, Senegal, Seychelles
Legal requirement to provide care to living donors in case of adverse or medical consequences	4	Algeria, Ethiopia, Senegal, Seychelles
Prohibition of organ trafficking/transplant commercialization	9	Algeria, Burkina Faso, Comoros, Côte d’Ivoire, Mali, Namibia, Nigeria, Rwanda, Senegal
Legal permit and regulation of financial incentives for living donors	0	None
Import or export of organs authorized	3	Ghana, Namibia, Rwanda
Import or export of organs explicitly prohibited	3	Algeria, Burkina Faso, Seychelles
Legal requirements for organ and tissue donations from living donors^a^	11	Algeria, Burkina Faso, Comoros, Côte d'Ivoire, Kenya, Mali, Nigeria, Rwanda, Senegal, Seychelles, Uganda
**No. of countries having an organization and management system**		
Authorization for transplant services	13	Algeria, Burkina Faso, Comoros, Côte d'Ivoire, Ethiopia, Ghana, Guinea, Kenya, Madagascar, Mali, Senegal, Uganda, Zimbabwe
Ethics Committees at the national or local level	11	Algeria, Burkina Faso, Comoros, Côte d’Ivoire, Ethiopia, Gabon, Kenya, Mali, Nigeria, Rwanda, Senegal
Government recognized authority at the national level	7	Algeria, Côte d’Ivoire, Ethiopia, Ghana, Mali, Senegal, Uganda
Setting up protocols, guidelines, recommendations	6	Algeria, Comoros, Côte d'Ivoire, Ethiopia, Mali, Senegal
Transplant follow-up registries for post-transplant living donor and for recipients	5	Algeria, Côte d'Ivoire, Ethiopia, Namibia, Uganda
Affiliation with an international organ allocation organization	0	None
Cooperation framework to allow transplantation abroad	6	Algeria, Côte d’Ivoire, Ethiopia, Kenya, Namibia, Uganda
Training programme for staff in place	2	Côte d’Ivoire, Ethiopia
**Source of funding^b^**		
Public	6	Algeria, Comoros, Ethiopia, Ghana, Mali, Seychelles
Private	1	Côte d’Ivoire
Public and Private	2	Namibia, Uganda
Not specified	5	Eswatini, Gabon, Kenya, United Republic of Tanzania, Zimbabwe

^a^ Includes written consent of the donor, ethical committee approval, psychiatric evaluation of the donor, judicial approval and external medical advice.

^b^ Only 14 countries provided information about funding.

## Legal and regulatory frameworks

We found that some countries had legal requirements in place for organ and tissue donations from living donors, such as written consent and ethical committee approval. Eleven countries have a law prohibiting organ trafficking, nine countries have banned transplant commercialization and three countries forbid organ import and export. Most countries did not yet establish a specific or comprehensive legislation. For instance, some legal requirements were in place only for living donors, but not for recipients and deceased donors. Among these countries, governments intended to adopt new legal requirements in eight countries. In Kenya, the health ministry had drafted new legislation, which covers the donation of organs and tissues from both living and deceased donors. 

The weak regulatory frameworks observed in these countries are often insufficient to ensure the effective regulatory oversight needed for the implementation of quality standards for organ transplantation. The assessment showed that transplantation activities in the region are in its infancy and solid legal and regulatory frameworks need to be established. The development and implementation of such frameworks can contribute to mitigate the major concern of the commercial transaction involved in most live unrelated donors for renal transplants in low- and middle-income countries.^20^^,^^22^

## National capacity

We only identified 62 transplant centres across seven countries in the region, including centres for kidney, heart, cornea, liver and bone marrow transplantations. Survey participants responded that, in general, the need for transplantation was important for patients, but the issue was the consent of a living donor for organ donation. We noted that about 350 kidney transplants from living donors were performed in four countries with transplantation centres (Table 2). In contrast to other WHO regions, kidney transplantations from deceased donors were not performed in the region, except in South Africa.

**Table 2:** Kidney transplant activities per country having reported functional programmes in the WHO African Region

Country	No.	Activity
Côte d’Ivoire	15	Single kidney transplants from related living donors
Ethiopia	12	Single kidney transplants (8 from related living donors and 4 from unrelated living donors)
Kenya	45	Kidney transplants from related living donors
Nigeria	> 270	At least 120 kidney transplants from related living donors and at least 150 from unrelated living donors

In the few countries having transplant centres, the national programmes donation and transplantation of organs and tissues were not consolidated. Indeed, these programmes had generally inadequate infrastructures, insufficient institutional support, lack of technical expertise, including competent human resources and technology. In addition, most transplant centres are located in major urban centres or capital cities, which could be an access barrier to treatment. Furthermore, the lack of public education, awareness and motivation for organ donation in most countries, especially in those with many religious, cultural, and social traditions, can create barriers to access donation and transplantation services.

## Organization and management

Effective organization and management are key elements to successful transplantation programmes. The assessment showed that most countries with a transplantation programme have not yet established functional coordination mechanisms for the programmes. Examples of these functions are: authorization for transplant services; ethics committees and government recognized national authority; setting up protocols, guidelines; recommendations for health-care workers and national health authorities; and transplant follow-up for post-transplant living donors and recipients. In addition, the treatment is not yet sufficiently integrated into national health development programmes and the collaboration between African countries is limited. Only seven countries (Algeria, Côte d’Ivoire, Ethiopia, Ghana, Mali, Senegal and Uganda) have established authorities at the national level responsible for overseeing transplant activities. In Kenya, a department integrating the national management of tissues, organs and blood transfusion was created in 2018. Furthermore, since the demand is higher than the supply, six countries (Algeria, Côte d’Ivoire, Ethiopia, Kenya, Namibia and Uganda) established a cooperation framework or agreement with partners to allow patients to be transplanted abroad. 

We found an insufficient multisectoral response to organ and tissue donations and transplantations, such as public education and awareness campaigns, engaging other development sectors in addressing transplantation issues and mobilizing adequate resources in the region. Other sectors, except health, had little involvement in transplantations issues. The assessment also revealed an absence of a strategic guidance at the regional level.

One of the challenges we identified for the establishment of transplantation programmes in the African Region was the limited data to monitor organ and tissue transplants to inform policy-making. Indeed, most countries did not include performance indicators related to organ and tissue donation and transplantation in their national health information systems. In addition, these countries did not conduct surveys to collect data on donations and transplantations, leading to a lack of accurate, reliable and real-time information in the region.

## Economic aspects

The relatively modest expenditure on health care by governments, seen in most low- and middle-income countries, contributes to the low transplantation activity. In addition to the legal, technical and organizational issues, the high cost of organ transplantations and immunosuppression therapies, coupled with inadequate financial coverage in most countries, are also barriers to equitable access to these medical procedures. The assessment showed that financial resources for organ and tissue donations and transplantations came from public sources in six countries (Algeria, Comoros, Ethiopia, Ghana, Mali and Seychelles) while recipients were responsible for paying for post-transplant care and drugs in 14 countries (Burkina Faso, Côte d'Ivoire, Ethiopia, Gabon, Ghana, Guinea, Kenya, Madagascar, Mali, Namibia, Nigeria, Rwanda, Uganda and Zimbabwe) including for procedures done outside these countries. Furthermore, living donors had to pay for follow-up care in eight countries (Burkina Faso, Gabon, Guinea, Kenya, Madagascar, Namibia, Uganda and Zimbabwe). Only in Algeria, all transplant recipients had access to immunosuppressive agents and other medicines free of charge.

To improve financial risk protection and access to high quality and safe organ donations and transplantations, the Abuja Declaration, which urged countries to allocate at least 15% of their annual budget to the health sector, should be effectively implemented to contribute to the attainment of universal health coverage.^23^

## Conclusion

The present assessment provided an overview of the current situation of organ and tissue donations and transplantations in the African Region and can serve as the baseline for WHO support to countries. The findings indicate a large variation between countries in terms of their capacity to provide for patients who need this type of treatment. The limited overall capacity for organ and tissue donations and transplantations in the region calls for consolidated efforts to strengthen legal frameworks and management and monitoring systems, as well as funding. Our results show that countries in the WHO African Region have a long way to go in meeting the goals set in the *WHO guiding principles on human cell, tissue and organ transplantation* and related WHA resolutions.^9^^–^^11^

Indeed, the region faces several key challenges, most of which can be met by (i) strengthening legal and regulatory frameworks of organ and tissue donation and transplantation; (ii) investing in strengthening currently weak health systems to ensure that donors and recipients receive high quality care during the transplant procedure, as well as follow-up care; (iii) boosting the regional capacity through the international cooperation and intercountry learning, including the development of a national strategy for donation and transplantation of human organs and tissues.
